# Functional analysis of the *Hsf4^lop11^* allele responsible for cataracts in *lop11* mice

**Published:** 2011-11-23

**Authors:** Lina Liang, Ryan Liegel, Brad Endres, Adam Ronchetti, Bo Chang, D.J. Sidjanin

**Affiliations:** 1 Department of Cell Biology, Neurobiology and Anatomy, Medical College of Wisconsin, Milwaukee, WI; 2 Human and Molecular Genetics Center, Medical College of Wisconsin, Milwaukee, WI; 3 The Jackson Laboratory, Bar Harbor, ME

## Abstract

**Purpose:**

Lens opacity 11 (*lop11*) is a spontaneous autosomal recessive mouse mutation resulting in cataracts. Insertion of an early transposable element (ETn) in intron 9 of heat shock factor 4 (*Hsf4*) was previously identified as responsible for *lop11* cataracts. Although molecular analysis showed that the ETn insertion resulted in an aberrant *Hsf4* transcript encoding a truncated mutant HSF4^lop11^ protein, the function of the mutant HSF4^lop11^ protein was not investigated. The goal of this study was to functionally evaluate the mutant HSF4^lop11^ protein and to establish the onset and progression of cataracts in *lop11* lenses.

**Methods:**

HSF4 is expressed as two alternatively transcribed isoforms *Hsf4a* and *Hsf4b.* Given that only *Hsf4b* is expressed in the lens we pursued evaluation of the mutant *Hsf4b* isoform only. Recombinant wild type HSF4b and mutant HSF4b^lop11^ proteins were analyzed using elecrophoretic mobility shift, reporter transactivation, western blotting and protein half-life assays in HEK293 cells. Prenatal and postnatal wild type and *lop11* lenses were evaluated using a combination of clinical, histological, and immunohistological analyses.

**Results:**

HSF4b^lop11^ stability and nuclear translocation of did not differ from wild type HSF4b. However, HSF4b^lop11^ exhibited abolished HSE-mediated DNA binding and transactivation. Further investigation identified that HSF4b^lop11^ fails to form trimers. Histological analysis of *lop11* lenses indicated the persistence of nuclei in lens fiber cells as early as postnatal day 0.5 (P0.5). No differences were observed between wild type and *lop11* in lens epithelial cell proliferation and spatio-temporal differentiation to fiber cells. However, by P10–12, *lop11* lenses develop severely vacuolated cataracts commonly accompanied by rupture of the lens capsule and release of the lenticular material in the vitreous cavity. Clinically, *lop11* vacuolated cataracts were visible upon eyelid opening between P12–14.

**Conclusions:**

The ETn insertion in *lop11* mice results in abolished HSF4b function. Loss of 132 amino acids from the COOH-terminus in HSF4b^lop11^ results in the failure of trimer formation and subsequent failure of HSE-mediated DNA binding and transactivation. These findings highlight the importance of the COOH-terminal region for normal function. The persistence of nuclei in postnatal *lop11* lens fiber cells was identified as the initial lens abnormality, thus confirming a previously identified role of HSF4b in denucleation of lens fiber cells. By P14 *lop11* lenses develop severe fiber cell vacuoles although how the loss of HSF4b function results in this process remains unknown. Collectively, these findings further our understanding of the mechanism of HSF4 loss of function as well as the resulting implications on *lop11* cataractogenesis.

## Introduction

Heat shock transcription factor 4 (HSF4) belongs to a family of highly evolutionarily conserved heat shock transcription factors (HSFs) that control gene expression in response to environmental and developmental stress conditions [1,2]. Proteins of the HSF family are characterized by a highly conserved winged helix-turn-helix domain [3] that mediates binding to a heat shock element sequence composed of at least three inverted repeats of consensus nGAAn sequence [2,4]. In addition to the winged helix-turn-helix domain, HSFs contain a leucine zipper-like heptad repeat A and B (HR-A/B) that facilitates trimerization of the HSF monomers [5]. It has been established that trimerization of HSFs is a prerequisite for DNA binding [5]. Most members of the HSF family of proteins also contain and an additional leucine zipper-like domain (HR-C) that suppresses trimer formation [6]. Under low-stress conditions HSFs stay as monomers or dimers and upon stress-mediated activation they are converted into trimers which subsequently bind the heat shock element [7]. Unlike other HSFs, HSF4 does not contain a HR-C domain and is thus a constitutive trimer capable of binding DNA. HSF4 is expressed as two alternatively spliced variants, *Hsf4a* and *Hsf4b,* which only differ in the inclusion of an additional 30 amino acids in HSF4b [8]. Functional analyses have shown that HSF4a acts as a transcriptional inhibitor and HSF4b acts as a transcriptional activator [8,9]. Interestingly, HSF4b can also act as a transcriptional repressor depending on the cell type and target genes [8,9]. The regulation of either HSF4 isoform is not yet clear, although phosphorylation and sumoylation have been implicated [10,11].

Genetic studies have unequivocally established that HSF4 plays an essential role for the transparency of the ocular lens. Mutations in *HSF4* have been identified in families with both autosomal dominant and recessive forms of hereditary congenital cataracts [12-17]. However, the molecular basis of the autosomal dominant and autosomal recessive modes of inheritance associated with *HSF4* mutations is not well understood. Targeted deletion of *Hsf4* in mice results in cataracts [9,18,19],  confirming a critical role of HSF4 for normal lens function. Analysis of lenses from *Hsf4^−/−^* mice identified defects in proliferation, differentiation and maturation of lens fiber cells [9,18,19] as well as altered expression of heat shock proteins, crystallins and intermediate filaments such as vimentin [20]. In the mouse and human lenses only the *Hsf4b* form is expressed, suggesting an essential role of the HSF4b isoform in maintaining lens transparency.

Lens opacity locus 11 (*lop11*), a spontaneous mouse mutation resulting in autosomal recessive cataracts, was previously mapped to mouse chromosome 8. Genetic analysis identified an early transposable element (ETn) in intron 9 (g.IVS9–61insETn) of *Hsf4* [21]. The same mutation was also identified in the lens disrupter 1 (*ldis1*) cataract mouse previously mapped to mouse chromosome 8 [21,22]. The ETn insertion acts as a pseudo-exon resulting in a chimeric transcript composed of *Hsf4* exons 1–9, the ETn pseudo-exon and a premature stop codon within the ETn sequence resulting in truncation of the remaining 3′ end of *Hsf4*. While the mutation was identified, the molecular consequences of the ETn insertion on the HSFb protein function had not yet been investigated. The goal of this study was to functionally evaluate the HSF4 mutant protein from *lop11* mice. Given that only the *Hsf4b* isoform is expressed in the lens, our focus was on the functional assessment of the HSF4b isoform. Our results show that the ETn insertion in *lop11* mice leads to a loss of HSF4b function, defects in postnatal lens cell fiber maturation and vacuolated cataracts.

## Methods

### Clinical and histological evaluation

The *lop11* allele was maintained on a mixed RIIIS/J X Cast/EiJ X C57BL/6 background. C57BL/6 mice obtained from the Jackson Laboratory (Bar Harbor, ME) were used as controls. Both wild type C57BL/6 and *lop11* mice were examined with a Topcon SL-D8Z slit lamp ophthalmoscope with a Nikon dSLR-based Photo Slit Lamp imaging system following mydriasis with 1% Atropine Sulfate (Bausch & Lomb, Rochester, NY). At least eight mice per age group were clinically examined. For histological analysis of lens morphology, eyes from wild type and *lop11* mice were collected at the embryonic days: E14.5, E16.5, and postnatal days: P0.5, P7, P10, P12, P14. They were subsequently fixed in 4% paraformaldehyde (Richard-Allan Scientific, Kalamazoo, MI), embedded in paraffin, cut to 4 µm sections and stained with hematoxylin and eosin (H&E) as previously described [23]. All lens sections in histological and immunological studies were cut through both the pupil and optic nerve to ensure comparable representation of the lens.

### DNA clones, cell cultures, and transfections

The wild type mouse *Hsf4b* (AB029349) and mutant *Hsf4b^lop11^* cDNAs were PCR-cloned into the pcDNA4/HisMax TOPO® TA Expression vector (Invitrogen, Carlsbad, CA) containing an in-frame NH_2_-terminal His tag using the following primers: *Hsf4* Fwd 5’-CAG GAA GCG CCA GCT G-3’, *Hsf4* WT Rev 5’-TCA GGG AGA GGA GGG ACT GG-3’, and *Hsf4*^lop11^ Rev 5’-TCA GGT TCC AGC AGT GGG C-3’. The HSF-TK-luciferase reporter was generated by PCR-amplifying a 500 bp fragment upstream of the start site of αB-crystallin (NM_009964) containing a HSE element using the following primers, incorporating 5’-SacI and 3’-XhoI restriction sites: HSE Fwd 5’-CAA CAA GAG CTC CCA GTC AGA CAC CTA GTT CTG CTC TC, and HSE Rev 5’-GTT GTT CTC GAG GTG GCT AGA TGA ATG CAG AGT CG-3’. The PCR fragment was initially TA-cloned into a pDrive cloning vector (Qiagen, Valencia, CA) and the insert was cut out with SacI and XhoI restriction nucleases and subsequently cloned into SacI and XhoI sites of pGL3Basic using standard protocols. Plasmids were purified with the QIAGEN Plasmid Midi Kit (Qiagen). HEK293 cells were obtained from ATCC (Manassas, VA) and cultured as suggested by the supplier. All transfections were carried with Lipofectamine^TM^ LTX (Invitrogen) using the manufacturer’s cell-line-specific protocols.

### Luciferase assay

About 1.25×10^5^ HEK293 cells per well were seeded in 24-well dishes in complete media containing 10% FBS-MEM for 16 h and then transfected with 300 ng/well of effector DNA, 110 ng/well reporter DNA, and 60 ng/well pcDNA_lacZ (for normalization of transfection efficiency). Following transfection, the medium was replaced with 1 ml of fresh 10% FBS-MEM and incubated for an additional 24 h after which cells were lysed using the Luciferase Assay System (Promega, Madison, WI). β-galactosidase activity was measured in lysates using the β-galactosidase Assay System (Promega, Madison, WI). Luciferase activity was subsequently normalized relative to β-galactosidase activity. All luciferase assays were performed a minimum of three times, each time in triplicate.

### Electrophoretic mobility shift assay (EMSA)

HEK293 cells were seeded at 1.6×10^6^ cells/100 mm dish in complete media containing 10% FBS-MEM for 16 h and then transfected with 14 μg of either wild type *Hsf4* or *Hsf4^lop11^* clones. Following transfection, the medium was replaced with 12 ml fresh MEM + 10% FBS and cells were incubated for an additional 24 h. Nuclear and cytoplasmic extracts were collected using the CelLytic NuCLEAR extraction kit (Sigma, St Louis, MO). Oligonucleotide labeling, binding, and the electrophoresis using the Gel Shift Assay System (Promega) were used following the manufacturer’s protocol. The oligonucleotide 5′-TGA CCT CAC CAT TCC AGA AGC TTC AGA AGA-3′ containing the HSE motif [24] (underlined) was used for the binding assay. For oligonucleotide labeling, ATP [γ-^32^P]- 3000 Ci/mmol 10 mCi/ml was used following manufacturer’s protocol. To determine specificity of the HSE binding reaction, unlabeled HSE oligo, unlabeled SP1 5′-ATT CGA TCG GGG CGG GGC GAG C-3′ and 1 µl of mouse monoclonal Anti-His G antibody (Invitrogen) were individually added to the reaction mix. All binding reactions were incubated at RT for 20 min and electrophoresed at RT in 1× TAE on a 4% acrylamide 40:1 acrylamide:bisacrylamide gel for 1 h at 250 V . Following electrophoresis, gels were transferred onto 3M paper, covered with plastic wrap, dried on a gel drier and exposed to X-ray film at −70 °C for 30 min.

### Protein stability

HEK293 cells were seeded at 1.6×10^6^ cells/100 mm dish in complete media containing 10% FBS-MEM for 16 h and then transfected with 14 μg of either wild type *Hsf4* or *Hsf4^lop11^* clones Following transfection, the medium was replaced with 12 ml fresh MEM + 10% FBS and incubated for an additional 24 h at which time the cells were divided into 24 well plates. Cells were incubated with cycloheximide (20 mg/ml) for 0 (DMSO vehicle only), 0.5, 1, 2, 4, and 6 h, lysed and subjected to western blotting. Signals from western blot analysis were analyzed with Photoshop software (Adobe Systems, San Jose, CA) and plotted using data from three independent experiments.

### Western blots

Western blots were performed as previously described [21] using an anti-His G monoclonal antibody (Invitrogen). The detection was performed an ECL western blotting detection kit (Amersham Biosciences, Piscataway, NJ) as previously described [21]. Briefly, cell lysates were electrophoresed on a 10% acrylamide gel and transferred to a PVDF membrane which was then blocked using 5% milk in PBST for 1 h, incubated at 4 °C with primary antibody overnight, washed 3× in PBST, incubated at RT for 1 h with an HRP-conjugated secondary antibody in PBST, washed 3× in PBST, treated with ECL reagent, and exposed to film.

### BrdU assay and immunohistochemistry

In proliferation studies, pregnant mice at P16.5 were injected intraperitoneally with 100 µg/g of bodyweight with 5-bromo-2’-deoxyuridine (BrdU; Invitrogen). After 2 h, pregnant mice were sacrificed, the embryos were collected and paraffin processed as described above. BrdU detection was performed using the BrdU Staining Kit (Invitrogen) following the manufacturer’s protocol. The BrdU-labeling index was determined as (BrdU-positive epithelial cells/total epithelial cells) × 100%.

Major intrinsic protein of eye lens fiber (MIP) immunostaining was performed using rabbit Anti-Aquaporin0 (Calbiochem, Gibbstown, NJ) and Alexa Fluor 488 conjugated goat anti-rabbit IgG, (Invitrogen). E-cadherin immunostaining was performed using rabbit anti-E-cadherin antibody (Cell Signaling Technology, Danvers, MA) and the Rabbit UniTect ABC Kit (Calbiochem) following the manufacturer’s protocol. Following immunostaining, nuclei were stained with 4’,6’-diamidino-2-phenylindole (DAPI) or TO-PRO3 (Invitrogen) using standard methods and the slides were mounted with Acrytol Mounting Medium (Surgipath Company, Richmond, IL) or with Vectashield mounting medium (Vector Laboratories, Burlingame, CA). All slides were visualized with either a Nikon Eclipse 600 Fluoroscope (Tokyo, Japan) or Leica Microsystems SP2® Laser Scanning Confocal Microscopic Imaging System (Wetzlar, Germany).

## Results

### HSF4b^lop11^ mutant protein

**Figure 1:**
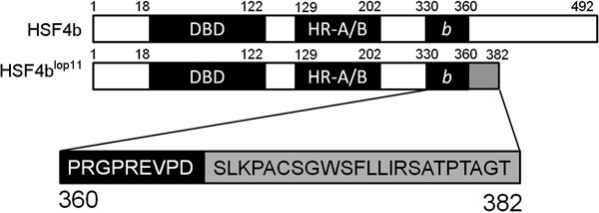
Wild type HSF4b (BAA84583.1) and putative HSF4b^lop11^ proteins. A schematic of HSF4b conserved domains: the DNA binding domain (DBD), the oligomerization domain (HR-A/B), the 30 amino acids HSF4b isoform domain (*b*) and the 23 amino acids originating from the ETn insertion (gray box). The ETn insertion results in a premature stop and a loss of 132 amino acids from the COOH-terminal domain. Numbers on the top of each figure represent the amino acid residues.

In *lop11* mice, an ETn insertion was identified in intron 9 of *Hsf4* which results in a transcript consisting of *Hsf4b* exons 1–9, a pseudo-exon from the ETn sequence and a premature stop [21]. The chimeric transcript encodes a putative HSF4b^lop11^ (p.D360_R361ins23X) mutant protein in which the first 360 amino acids are encoded by *Hsf4b* exons 1–9, followed by a stretch of 23 amino acids encoded by the ETn pseudo-exon and a premature stop (Figure 1). BLAST analysis of the 23 amino acid stretch encoded by the ETn pseudo exon did not identify significant homology with any known protein (not shown). Following the pseudo exon, the premature stop codon resulted in truncation of HSF4b and a loss of 132 amino acids from the COOH-terminal end (Figure 1). The ProtParam tool predicted that the His-tagged HSF4b would have a mass of 57 kDa whereas the mutant His-tagged HSF4b^lop11^ would have a mass of 45 kDa.

### HSF4b^lop11^ nuclear trafficking, DNA binding, and transactivation

**Figure 2:**
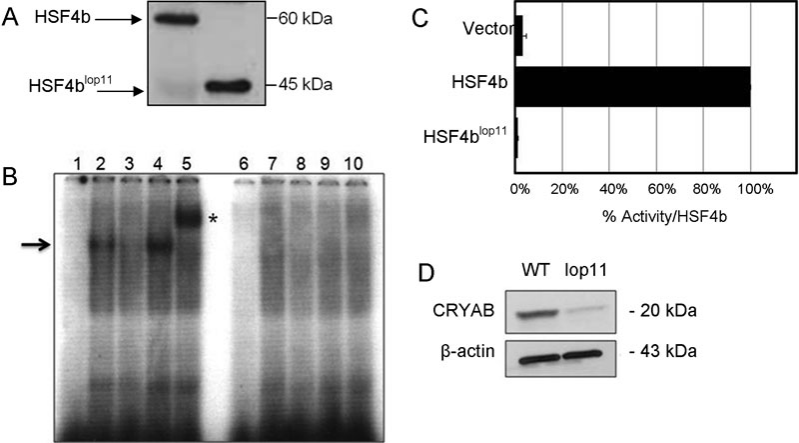
Analysis of wild type HSF4b and mutant HSF4b^lop11^ proteins. **A**: western blot of nuclear extracts transfected with wild type *Hsf4b* or *Hsf4b^lop11^* clones. **B**: EMSA showed absence of HSE-DNA binding of HSF4b^lop11^ when compared to wild type HSF4b. A negative control was a binding reaction with nuclear extracts following transfection with an empty vector (pcDNA3.1; lanes 1 and 6). Labeled HSE and wild-type HSF4b (lane 2) form a complex (arrow). Specificity of HSF4b binding to HSE was determined by a specific competition with cold HSE (lane 3), nonspecific competition by addition of unlabeled SP1 (lane 4) and supershift (asterisk) by addition of His-antibody (lane 5). Nuclear extracts from cells transfected with *Hsf4b^lop11^* did not result in HSE-DNA binding in the presence of labeled HSE (lane 7), presence of labeled and unlabeled HSE (lane 8), presence of labeled HSE and unlabeled SP1 (lane 9) and labeled HSE and His-antibody (lane 10). **C**: Transactivation of HSE-reporter in HEK293 cells by wild type HSF4b and HSF4b^lop11^ proteins. Luciferase values were normalized to β-galactosidase activity, averaged for three separate transfections and expressed relative to the ration for wild-type *Hsf4b.* Error bars represent SEM. Asterisk indicates samples with a significant difference (p<0.05; *t-*test) calculated from comparison with wild-type. **D**: western blot analysis of lens protein extracts from P7 wild type (wt) and *lop11* lenses immunobloted with CRYAB-antibody (top panel) showing severely reduced CRYAB levels in *lop11* lenses. The molecular mass is indicated to the right of each blot. Even loading was confirmed by immunobloting with β-actin (bottom panel).

As an initial step, we wanted to investigate if the mutant HSF4b^lop11^ protein is properly trafficked into the nucleus. The location of a nuclear localization signal (NLS) within HSF4b has not yet been identified. In-silico analysis of human and mouse HSF4b by PredictProtein, an automated tool for the analysis of protein feature prediction, did not identify any putative NLS sequences. Therefore, we set out to experimentally evaluate if HSF4b^lop11^ is trafficked into the nucleus. Both wild type *Hsf4b* and *Hsf4b^lop11^* chimeric transcripts were cloned into a vector containing an NH_2_-terminal His-tag and transfected into HEK293 cells. Western blotting identified anti-His immunoreactive bands for HSF4b and HSF4b^lop11^ in both nuclear (Figure 2A) and cytosolic lysates (not shown) similar in size as predicted by the ProtParam analysis. These findings indicated that trafficking of HSF4b^lop11^ to the nucleus was not compromised.

Next, we evaluated the ability of HSF4b^lop11^ to bind to a HSE motif by using an electrophoretic mobility shift assay (EMSA). It was previously shown that HSF4 binds to the HSE derived from the rat αB-crystallin (*Cryab*) promoter [25]. We identified the syntenic HSE consensus sequence in the promoter of the mouse αB-crystallin (NM_009964) and used it to assess the binding of Hsf4b^lop11^ in our EMSA assay. Our analysis showed that HSF4b formed a complex with the HSE sequence (Figure 2B). In contrast to HSF4b, nuclear extracts from cells transfected with HSF4b^lop11^ did not bind to the HSE sequence under any conditions (Figure 2B). Furthermore, we wanted to assess the transactivation potential of HSF4b^lop11^ via luciferase assay. A 500 bp fragment upstream from the start codon from the mouse αB-crystallin gene was cloned in a luciferase reporter vector. Transfection of the HEK293 cells with wild type *Hsf4b* caused an approximately 80 fold increase in activation of the reporter gene when compared with a transfection with an empty expression vector (Figure 2C). In contrast, transfection of the clone containing the *Hsf4b^lop11^* transcript showed a complete absence of any transactivation activity with the same reporter gene (Figure 2C). To assess if expression of CRYAB protein was also compromised in vivo, we performed western blot analysis on protein extracts from wild type and *lop11* lenses. Immunoblotting revealed severely reduced CRYAB levels in *lop11* lenses as normalized to β-actin (Figure 2D).

### HSF4b^lop11^ protein stability and trimerization

**Figure 3:**
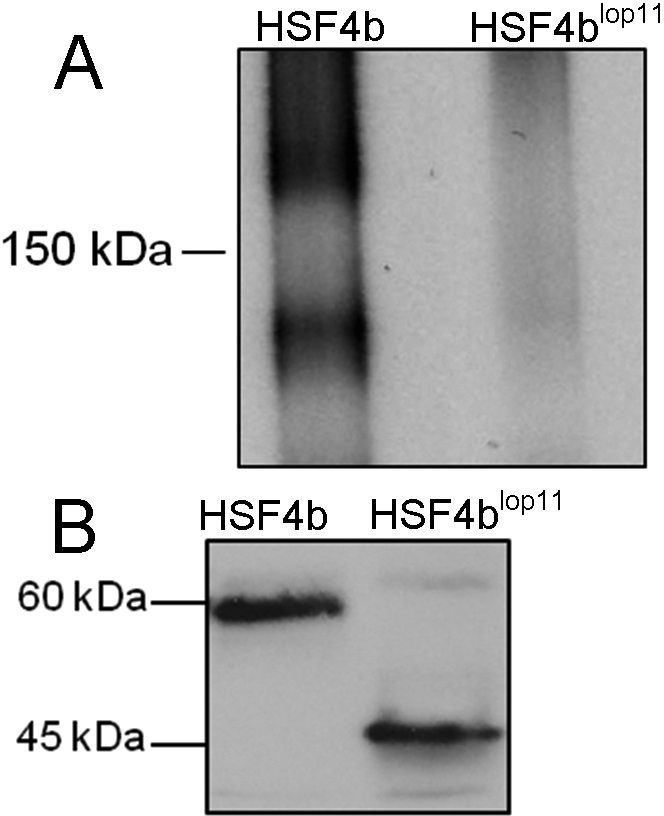
Comparison of the electrophoretic mobility of HSF4b and HSF4b^lop11^ proteins under non-denaturing and denaturing conditions. Nuclear extracts from cells transfected with *Hsf4b* and *Hsf4b^lop11^* were separated by **A**, non-denaturing PAGE or **B**, SDS/PAGE and immunoblotted with anti-His antibody. The homotrimers could only be detected with HSF4b (arrow) as show in **A**.The same nuclear extracts as shown in **A** when run under denaturing conditions exhibited presence of the monomeric HSF4b and HSF4b^lop11^ subunits as shown in **B**. The molecular mass is indicated to the left of each blot.

To further examine the molecular basis of the failure of the HSF4b^lop11^ to bind HSE and transactivate a HSE containing sequence, we examined the possibility that the mutant HSF4b^lop11^ was unstable. HEK293 cells transiently transfected with either *Hsf4* or *Hsf4b^lop11^* clones were treated with cyclohexamide to inhibit protein synthesis and assess the turnover rate of HSF4b and HSF4b^lop11^ proteins. No significant difference in stability was observed between HSF4b and HSF4b^lop11^ over a period of 4 h (not shown). We also examined the ability of the HSF4b^lop11^ to form trimers. Nuclear extracts from cells transiently transfected with *Hsf4b* or *Hsf4b^lop11^* were immunoboltted following non-denaturing gel electrophoresis. An anti-His immunoreactive band of about 180 kDa and 120 kDa representing HSF4b homotrimer and homodimer respectively was observed in cells transfected with *Hsf4b*. In contrast, no anti-His immunoreactive bands corresponding to the dimeric or trimeric forms were observed in *Hsf4b^lop11^* samples (Figure 3A). Western blot analysis of the same nuclear extracts following electrophoresis under denaturing conditions confirmed the expression of monomeric forms of both HSF4b and HSF4b^lop11^ (Figure 3B).

### The *lop11* cataract phenotype

**Figure 4:**
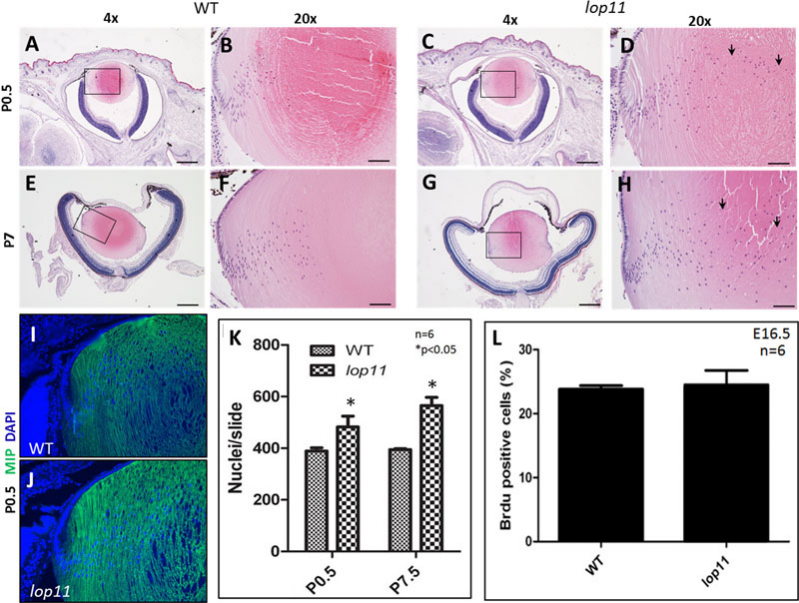
Characterization of *lop11* lenses. The persistence of nuclei in *lop11* lens fiber cells (arrows) was first observed in P0.5 lenses without any other significant morphological differences noted between wild type (**A, B**) and *lop11* (**C, D**) lenses; the same findings were noted in P7 wild type (**E, F**) and *lop11* (**G, H**) lenses. Quantification of lens fiber nuclei number in the lens cross-sections in P0.5 and P7.5 wild type and *lop11* lenses is shown in **K**. The error bars represent the SEM and asterisks indicate significant differences (p<0.05; *t-*test) calculated from comparison with wild-type. Immunohistochemistry of P0.5 wild type (**I**) and *lop11* (**J**) lenses using anti-MIP antibody showing lens fiber cell staining. **L**: BrdU incorporation in the lens epithelial cells of E16.5 mice. Percentages of BrdU-positive cells are shown from six different animals. Error bars represent SEM. The scale bars in **A**, **C**, **E**, and **G** indicate 250 µm, whereas **B**, **D**, **F**, and **H** are 50 µm. All sections are cut in the center of the lens, containing the pupil and optic nerve.

Histological analysis of cross sections from eyes at E16.5 and E18.5 did not identify any morphological differences between wild type and *lop11* lenses (not shown). At P0.5 and P7, *lop11* lenses appeared to exhibit a greater number of nuclei persisting in the lens fiber cells when compared to age-matched controls (Figures 4A-H). Quantification of lens fiber cell nuclei confirmed this finding (Figure 4K).

We also wanted to evaluate if premature differentiation of lens epithelial cells into the lens fibers played a role in greater number of nuclei observed in *lop11* lens fiber cells. In both wild-type and *lop11* lenses the expression of E-cadherin was restricted to the epithelium (not shown) and MIP expression was restricted to the cortical and nuclear fiber cells (Figure 4I,J). We also evaluated that the possibility that the increased number of nuclei observed in *lop11* lenses was associated with an increase in the lens epithelial cell proliferation. BrdU incorporation was evaluated in E16.5 wild-type and *lop11* lenses. No statistically significant difference in the number of BrdU positive cells in the *lop11* lens epithelium was observed when compared with age-matched lens epithelial cells from wild-type lenses (Figure 4L).

**Figure 5:**
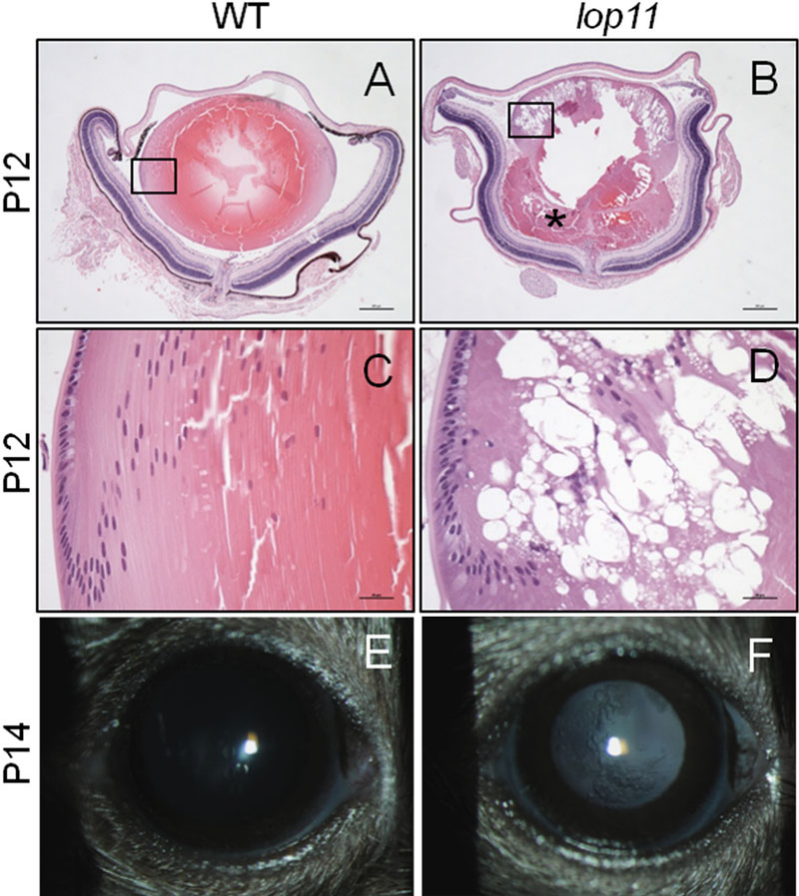
The *lop11* lens phenotype after P10. Between P10-P12 *lop11* lenses develop vacuolated cataracts that result in the rupture of the lens capsule (**B**) and release of the lentricular material in the vitreal cavity (asterisk). The boxed areas are depicted at the higher magnification in **C** and **D**. The vacuoles develop between cortical and nuclear fiber cells as shown in **D**. The *lop11* vacuolated cataracts are clinically visible following the eyelid opening between P12–14 (**F**) after which the cataract phenotype does not progress. In **A** and **B**, scale bars represent 200 µm; in **C** and **D** scale bars represent 20 µm.

Additional histological analysis further identified that between P10-P12 *lop11* lenses develop vacuoles present in the cortex and nucleus of the lens (Figure 5D). In addition, rupture of the lens capsule was present in the majority of *lop11* lenses, resulting in the release of lens material into the vitreous cavity (Figure 5B). Upon eyelid opening *lop11* vacuolated cataracts were clinically visible following mydriasis (Figure 5F). No further progression of the cataract phenotype was observed in older *lop11* mice (not shown).

## Discussion

In this study we show that the ETn insertion in intron 9 of *Hsf4* in *lop11* mice results in a truncated HSF4b^lop11^ protein that exhibits a loss of HSF4b function. The mutant HSF4b^lop11^ protein fails to form trimers and consequently exhibits abolished HSE-mediated DNA binding and transactivation. As a part of this study we established abolished transactivation of *Cryab*; we expect that expression of all HSF4b downstream targets such as heat shock proteins, crystallins, and intermediate filaments are altered in *lop11* lenses, as previously established for *Hsf4* null mice. The findings from this study also confirm previous findings showing that trimerization of HSF members is a prerequisite for the HSE-mediated DNA binding and transactivation [5]. HSF trimerization is mediated by an array of hydrophobic repeats within the trimer oligomerization domain (HR-A/B) [5]. In the mutant HSF4b^lop11^ protein, the HR-A/B domain is unaltered by the ETn insertion. However, HSF4b^lop11^ lacks 132 aa from the C-terminal end, suggesting that the absence of 132 aa from the COOH-terminal end in HSF4b^lop11^ most likely results in the failure to form trimers. We cannot exclude the possibility that the presence of the 23 amino acids encoded by the ETn pseudo exon at the COOH-terminal of HSF4b^lop11^ is responsible for the failure of HSF4b^lop11^ trimer formation. In comparison of our data in with recessive human mutations, we consider this less likely. In humans, four different mutations in *HSF4* result in autosomal recessive congenital cataracts [14-16]. Out of these, three mutations result in a loss of the COOH-terminal domain [14-16] and one mutation is within the HR-A/B trimerization domain [15]. The mechanism associated with recessive *HSF4* mutations has not yet been investigated. Our current hypothesis is that, in both human and mouse HSF4b, a loss of the COOH-terminal domain results in abolished trimerization and consequently abolished HSF4b function. Studies evaluating this hypothesis are the focus of our current investigation.

The functional analysis of HSF4b^lop11^ from this study also demonstrated that the COOH-terminal end is not involved in the trafficking of HSF4b to the nucleus. To date, no NLS elements in either human or mouse HSF4 have been identified or characterized. Our results from this study suggest that in mouse HSF4b a NLS is encoded within the first 360 amino acids, but the precise identification of the NLS requires further investigation. In two other members of the HSF family, HSF1 and HSF2, two bipartite type NLS sequences were identified in regions flanking the HR-A/B domain [26,27]. Although a great deal of conservation exists within the members of the HSF family, sequences flanking the HR-A/B domain differ between various HSFs. Therefore, the NLS sequence within HSF4b still remains elusive. Interestingly, in HSF1 and HSF2, the COOH-terminal domain contains two regulatory domains: one that acts as a transactivation domain under stress conditions and another one that represses the COOH-terminal activation domain in the absence of stress [27,28]. At this point it is unclear if the COOH-terminal domain of HSF4b also contains domains that may play a role in transactivation. Further functional dissection of HSF4b domains is needed to establish the essential regulatory domains for proper HSF4b function.

In examining the biologic consequences of HSF4b loss of function in *lop11*, histological analyses of postnatal *lop11* lenses identified the persistence of nuclei in lens fiber cells as the initial morphological abnormality associated with the mutation. Evaluation of lenses from three other strains of *Hsf4 null* mice also showed persistence of nuclei in postnatal lenses [9,18,19]. During normal development, lens epithelial cells undergo proliferation, followed by cell cycle withdrawal and terminal differentiation into lens fiber cells [29]. As lens fibers begin to mature, the nucleus and organelles are degraded via a highly regulated process resulting in an “organelle-free-zone” within the lens nucleus which is essential for lens transparency [30,31]. The mechanisms associated with organelle degradation are largely elusive [30,31]. Our findings and findings from others suggest that HSF4b functions in the denucleation process of lens fiber cells. However, it should be noted that mutations in other genes such as connexins, γ-crystallins, and DNase II-like acid DNase (DLAD) also show altered denucleation process [32-35]. Expression profiling of lenses from *ldis1* mice that carry the same ETn insertion in *Hsf4* as *lop11* mice, showed that expression of γ-crystallins was severely reduced [22]. In fact, the expression of many other genes was also altered in HSF4 null lenses [18,19,22]. It has been recently shown that an increase in vimentin expression may be associated with defects in denucleation process observed in *Hsf4-*null mice [20]. Therefore, the defects observed in denucleation in *lop11* lenses may be an indirect effect as a consequence of altered expression of HSF4b transcriptional targets that play a direct role in the denucleation process.

Our analysis of the *lop11* lens phenotype identified vacuoles and rupture of the lens capsule as a severe phenotype presented around P10–12. This finding suggests that at this later stage the lens may be more sensitive to the HSF4b loss of function. We cannot exclude the possibility that subtle defects, in addition to persistence of nuclei in lens fiber cells identified before the onset of vacuoles, may be occurring throughout the *lop11* lens development. Results from this study did not identify any differences in lens epithelial cell proliferation or differentiation between wild type and *lop11* before the onset of vacuolated cataracts. Interestingly, analysis of one of the *Hsf4 null* mice identified increased proliferation and premature differentiation of the lens epithelial cells [9] and the analysis of lens epithelial cell in another *Hsf4 null* mice did not identify any differences in proliferation or timing of differentiation [18]. Vacuolated lenses were reported for all other *Hsf4 null* mice, although the onset of vacuoles varied between different strains of *Hsf4 null* mice [9,18,19]. The mechanism of how loss of HSF4b functions results in lens vacuoles remains unclear. Capsule rupture was reported as a phenotype in one strain of *Hsf4* null strains [18], for half of mice in another *Hsf4* null strain [9] and none for the third *Hsf4* null strain [19]. Such discrepancies between *Hsf4* null strains including *lop11* may be associated with differences in the genetic background. Alternatively, differences in the *Hsf4* targeting constructs as well as environmental effects and diet can also play a role in the phenotype variability. It will be important in the future to examine the direct phenotypic consequences of HSF4-loss as well as the contribution of genetic background, epigenetic factors, and environmental factors for the onset and progression of *Hsf4* associated cataracts.
